# Three-Dimensional Organotypic Systems for Modelling and Understanding Molecular Regulation of Oral Dentogingival Tissues

**DOI:** 10.3390/ijms252111552

**Published:** 2024-10-28

**Authors:** Emily Ming-Chieh Lu

**Affiliations:** Centre for Host-Microbiome Interactions, Faculty of Dentistry, Oral and Craniofacial Sciences, King’s College London, London SE1 9RT, UK; emily.lu@kcl.ac.uk

**Keywords:** three dimensional organotypic, dentogingival, periodontium, periodontitis

## Abstract

Three-dimensional organotypic models benefit from the ability to mimic physiological cell–cell or cell–matrix interactions and therefore offer superior models for studying pathological or physiological conditions compared to 2D cultures. Organotypic models consisting of keratinocytes supported by fibroblasts embedded in collagen matrices have been utilised for the study of oral conditions. However, the provision of a suitable model for investigating the pathogenesis of periodontitis has been more challenging. Part of the complexity relates to the different regional epithelial specificities and connective tissue phenotypes. Recently, it was confirmed, using 3D organotypic models, that distinct fibroblast populations were implicated in the provision of specific inductive and directive influences on the overlying epithelia. This paper presents the organotypic model of the dentogingival junction (DGJ) constructed to demonstrate the differential fibroblast influences on the maintenance of regionally specific epithelial phenotypes. Therefore, the review aims are (1) to provide the biological basis underlying 3D organotypic cultures and (2) to comprehensively detail the experimental protocol for the construction of the organotypic cultures and the unique setup for the DGJ model. The latter is the first organotypic culture model used for the reconstruction of the DGJ and is recommended as a useful tool for future periodontal research.

## 1. Introduction

The dentogingival junction is a complex and unique structure responsible for the attachment between the tooth and oral mucosa. The dentogingival epithelia consist of two main compartments: the junctional epithelium (JE), and gingival epithelium (GE), between which lies a transitional compartment, the sulcular epithelium (SE) [1]. The JE is characterised by a non-keratinised epithelial phenotype, while the GE possesses a keratinised tissue phenotype [1]. The epithelial tissues are supported by the underlying connective tissues, known as the lamina propria. A key event in the pathogenesis of periodontitis is the apical migration of the JE, which along with the loss of alveolar bone, cementum and periodontal attachment, is a hallmark of periodontitis [2]. Controlling this tissue migration may be central to tissue regeneration and ultimately, reversing periodontitis.

Histological recombination studies suggested that the gingival lamina propria, as a superficial connective tissue (SCT), provided the permissive stimulus for epithelial growth and differentiation [3,4], and thus was responsible for maintaining the gingival epithelial phenotype. By contrast, it was proposed that the deeper connective tissues (DCT), such as the periodontal ligament tissues or periosteum, either did not generate the signals necessary for growth and differentiation or produced inhibitory signals that led to an immature, atrophic-like epithelium [3,5], comparable to the phenotype resembling the JE. These studies, however, were not able to demonstrate the specific molecular signals central to epithelial regulation.

The construction of 3D organotypic cultures in more recent years has demonstrated the inductive influence of fibroblasts in specifying epithelial phenotype [6,7]. Using homogenous fibroblast cell populations, our group reinforced the concept that distinct fibroblast populations have differential effects in supporting epithelial growth [2]. Specifically, we showed that human gingival fibroblasts (HGFs) facilitated epithelial growth while the human periodontal ligament fibroblasts (HPDLF) did not. We also showed that the highly specific expression of the Wnt antagonist, SFRP4, in HPDLF was central to the inhibition of epithelial growth and therefore was important in maintaining the normal structure and function of JE observed in health [2].

A recent systematic review, however, has highlighted the lack of standardised protocols for the construction of oral gingival organotypic models [8]. Moreover, there is a lack of established in vitro models in the literature by which to study the junctional epithelium and neighbouring tissues. Therefore, this paper aims (1) to summarise the literature with respect to 3D organotypic cultures for modelling oral dentogingival tissues; and (2) to comprehensively detail the protocol for the construction of the organotypic model of the dentogingival junction (DGJ). This in vitro model is the first published work in the construction of the DGJ and has potential to be a useful tool in periodontal research.

## 2. Landmark Historical Transplantation and Recombination Studies

The tissue transplantation studies of the 1970s [4,9] and recombination experiments of the 1980s [3,10,11] showed that the nature of the connective tissues was responsible for the variations in dentogingival epithelial phenotypes.

The heterotopic transplantation of gingival, alveolar and palatal mucosa by Karring and co-workers [9] showed that tissue specificities were retained following transplantation. A subsequent study by the same group, where free connective tissue grafts from gingiva or alveolar mucosa were transplanted into areas of non-keratinised alveolar mucosa, demonstrated that the signals for epithelial growth and keratinisation originated from the underlying gingival connective tissues [4].

**Figure 1:**
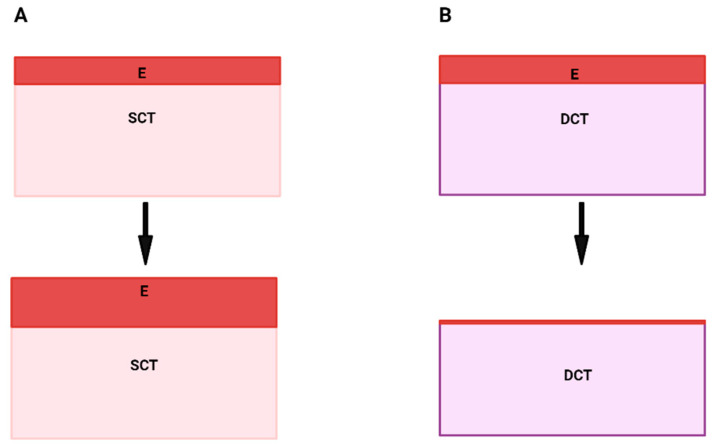
Diagram showing the differences in epithelial growth between an epithelium recombined with SCT versus DCT. When the epithelium is recombined with SCT, the epithelium will continue to increase in thickness; therefore, SCT supports epithelial growth (**A**). Conversely, when the epithelium is recombined with DCT, it fails to grow and atrophies over time (**B**). SCT = superficial connective tissue, DCT = deeper connective tissue, E = epithelium.

To reinforce this concept, seminal papers published in the 1980s demonstrated that epithelial growth was enhanced when the adult epithelium was recombined with the lamina propria or the dermis [3,12]. As SCTs, both the lamina propria and the dermis were thought to provide the permissive signals for epithelial growth and differentiation [3,12,13], Figure 1A. By contrast, when the adult epithelium was recombined with the DCT, the epithelium atrophied over time [12,13], Figure 1B, which suggested one of two possibilities: either DCTs did not provide the permissive signals for epithelial growth and keratinisation or that an active inhibitory signal was present within the DCT [5,11,14].

Clearly, these studies demonstrated that it was the connective tissues which were essential for the modulation of epithelial growth [15] and differentiation [5]. However, a partial phenotypic change in epithelial phenotype, as observed in some heterotypic recombination experiments, suggested that the connective tissues were not sending all of the signals required for complete morphologic differentiation [16,17,18], and that intrinsic influences derived from the epithelia also contributed to the overall epithelial morphogenesis [16,17].

Therefore, both the intrinsic and extrinsic influences from the underlying connective tissues were responsible for the regulation of the overall epithelial phenotype [3,4,5,11,13,14]. However, these studies were unable to demonstrate the cellular processes and the specific molecular signals responsible for the directive and inductive influences on the overlying epithelia.

## 3. Three-Dimensional Organotypic Cultures: General Principles

**Figure 2:**
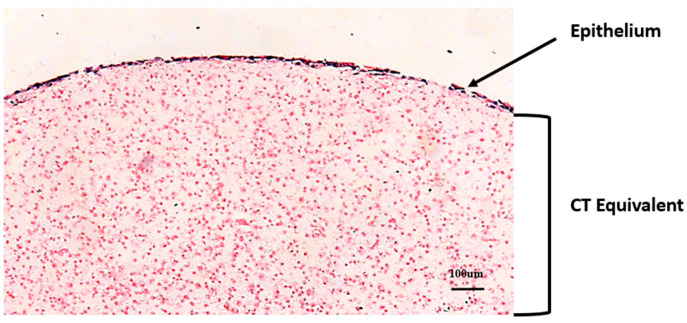
Haematoxylin and eosin section of a 3D organotypic construct, consisting of H400 epithelium cultured over collagen matrix enriched with PDL fibroblasts (connective tissue equivalent). The fibroblasts have been stained pink in this section. Image used with permission [19].

A 3D organotypic culture typically consists of keratinocytes supported by fibroblasts embedded in a connective tissue matrix (Figure 2) [19]. In comparison to 2D monolayer cultures, which fail to capture the complex physiological cellular/tissue interactions and processes [20,21,22,23], 3D organotypic cultures possess the ability to mimic complex physiological cell–cell and cell–extracellular matrix interactions [24]. Therefore, unlike 2D cultures, which offer simplicity, but do not represent the physiological cellular interactions and cell–extracellular matrix (ECM) interactions, 3D co-cultures are able to recapitulate the cellular function and physiological cell behaviour in vivo [25]. Alternatives to 3D organotypic models are the commercially available gingival mucosa (for example, MatTek https://www.mattek.com/reference-library/three-dimensional-polymer-scaffolds-for-high-throughput-cell-based-assay-systems/, accessed on 22 October 2024,), which have already been used to study a broad range of clinical applications, including investigations into host microbiome interactions [26,27]. However, the application for which the model is being used would dictate the selection of specific cells, medium and scaffold [28].

Three-dimensional organotypic cultures also have several advantages over animal models, such as the ability to study an isolated cellular interaction or molecular pathway without complex physiological factors present in an intact living mammalian model. Three-dimensional organotypic cultures provide an excellent alternative to animal models, and benefit from their reproducibility, which contrasts with the variations inherent in individual animals. These in vitro cultures enable the opportunity to study tissue interactions without ethical considerations, which would otherwise be necessary for animal studies or human clinical trials [26,27].

One of the fundamental differences between the 2D monolayer and 3D cultures is the presence of a substrate. Specifically, the physical and mechanical cues from the substrate will influence cellular behaviour via paracrine signalling between cell types. A critical step in the generation of 3D organotypic constructs is the culture at the air–liquid interface (ALI) [28]. Constructs are typically placed into Transwell inserts which contains a microporous membrane, allowing the passage of diffusible factors into the collagen matrix. Following a period where the constructs are submerged, the medium is removed from inside the Transwell and at the ALI, the constructs are then fed by the addition of media in the outer well. The oxygen tension created on the coronal surface of the cells results in keratinocyte differentiation, which makes the model clinically relevant [29], as it mimics the phenotype observed in the oral cavity [30].

Recent advances in tissue engineering have facilitated the development of models which better represent physiological systems. For example, the incorporation of immune cells or senescent fibroblasts in skin organotypic cultures has resulted in more meaningful models to study psoriasis, atopic dermatitis [31] and skin ageing [32], respectively. The ability of 3D cultures to model tissue fibrosis in the liver, kidney and lung [33] demonstrates their potential to provide a similar model for other chronic inflammation conditions, such as periodontal disease.

### 3.1. Paracrine Signalling of Fibroblasts Facilitates Keratinocyte Proliferation and Differentiation

Three-dimensional organotypic cultures enable the study of paracrine signalling between epithelial and fibroblast components under controlled in vitro conditions [7,16,17,18,34]. Several experiments have shown that direct contacts between keratinocytes and fibroblasts were not required to induce changes in the epithelial phenotype. For example, when the epithelium was separated from the dermis by a collagen gel, growth and maturation of the epithelium ensued [35].

Diffusible factors produced by fibroblasts were responsible for specifying and directing epithelial proliferation and differentiation [36,37]. In other words, the nature of the fibroblasts is important in providing the inductive signals for the specific epithelial phenotype.

Fibroblasts secrete a variety of diffusible factors such as keratinocyte growth factor (KGF), hepatocyte growth factor (HGF) and cytokines [38]. In particular, KGF is a major regulator of keratinocyte growth and differentiation [38]. HGF is a paracrine growth factor involved in the growth, acceleration of epithelial proliferation and preventing fibrosis [39]. The paracrine signalling of oral or gingival fibroblasts is thought to result in accelerated epithelial proliferation compared to dermal fibroblasts [20].

Some of the signals involved in epithelial proliferation and migration are controlled by the canonical Wnt pathway, which becomes activated in chronic inflammation and the wound healing of skin [40]. Specifically, canonical Wnt signalling enhances epithelial migration via a reduction in E-cadherin expression; and β-catenin promotes the production of ECM by fibroblasts, a process which is of fundamental importance for wound closure [40]. Crucially, the interactions between canonical Wnt, TGF-β and Notch signalling pathways are necessary for optimal wound healing [41]. These interactions are responsible for the secretion of soluble proteins that are involved in cell activation and transition through the different healing phases. For example, TGF-β promotes the differentiation of myofibroblasts via activation of the canonical Wnt pathway and thus mediates progression from the inflammatory phase to the remodelling phase of healing [41]. Notch signalling mediates the downregulation of Wnt signalling and thus a reduction in β activity, to promote cellular differentiation [42]. The activation of Notch signalling has been implicated in alveolar bone loss and an upregulation of TGF-β has been associated with periodontitis [43,44]. Conversely, it has been shown that a downregulation of Wnt activity is conducive with periodontal health (see Section 3.3), as reinforced by my group [2] and others [45,46,47].

Additionally, compared to skin fibroblasts, gingival fibroblasts secrete elevated levels of factors involved in inflammation and angiogenesis [20], as well as the production of higher levels of KGF, and HGF when co-cultured with keratinocytes [48]. Therefore, the paracrine signalling of these fibroblasts could be responsible for the accelerated rates of keratinocyte proliferation and differentiation, and thus faster wound healing compared with dermal keratinocytes [20,49].

### 3.2. Role of Distinct Fibroblast Populations in Supporting Epithelial Growth

**Figure 3:**
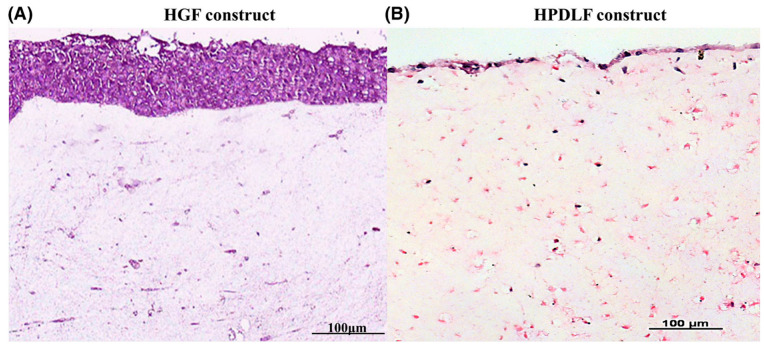
Haematoxylin and eosin sections of human gingival fibroblasts (HGFs) and human periodontal ligament fibroblasts (HPDLFs) demonstrating the differences in epithelial growth following a 21-day culture. Image used with permission from Wiley [2].

Organotypic cultures constructed using HPDLFs, HGFs, junctional and gingival epithelia showed that the specific epithelial phenotypes were determined and influenced by the underlying fibroblast subpopulation [7]. To reinforce this concept, a subsequent study showed that 3D organotypic cultures consisting of human gingival keratinocytes, that were either supported by HGFs or HPDLFs, were responsible for the development of the junctional or sulcular epithelia [6]. Recent findings from our group employing 3D organotypic cultures also echoed such findings [2]. We showed that HGFs supported a multi-layered epithelium, while HPDLF was associated with an atrophic epithelial phenotype (Figure 3) [2]. This suggests that the gingival lamina propria was responsible for the development and maintenance of the gingival epithelial phenotype, while the PDL tissues provided the non-permissive influences on the JE, thus sustaining a non-migratory and non-differentiating phenotype [2]. Therefore, these studies highlight the fundamental role of fibroblasts in influencing epithelial phenotype.

### 3.3. Molecular Regulation of the Dentogingival Epithelia

**Figure 4:**
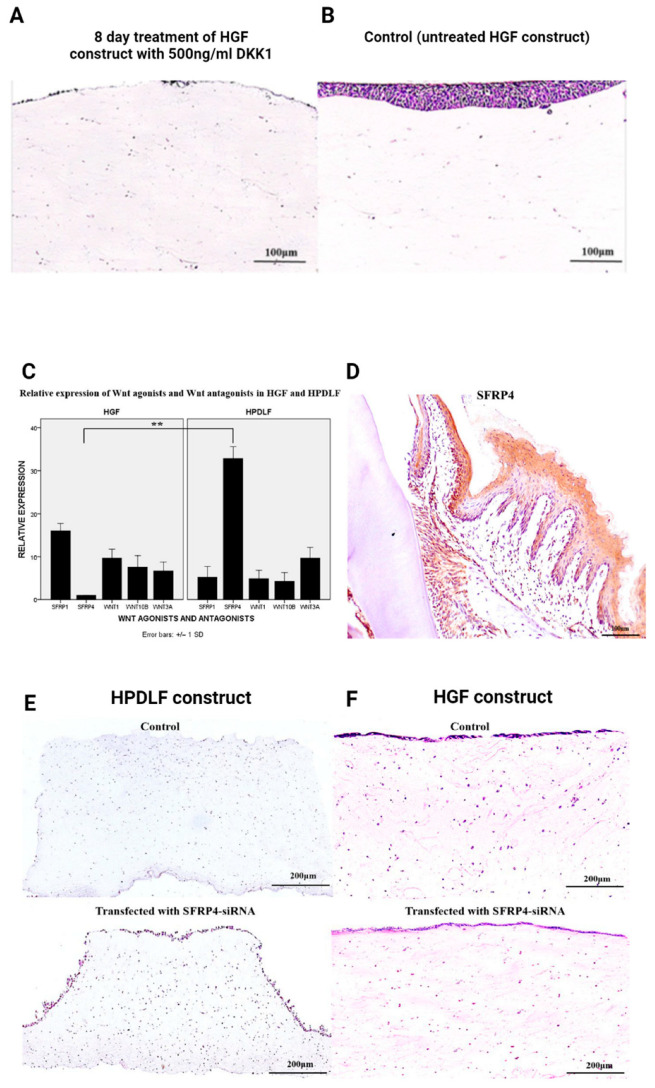
H&E sections showing the reduction in epithelial growth of HGF construct following treatment with 500 ng/mL DKK1 over 8 days (**A**) compared to control which showed progressive epithelial growth (**B**). Following testing for a range of Wnt agonists and antagonists, *SFRP4* was identified to be differentially expressed in HPDLFs (**C**) and this was reinforced by the proteomic expression of SFRP4 in the PDL tissues (**D**). H&E sections of constructs transfected with siRNA-SFRP4 (**E**,**F**). Treatment of HPDLF constructs with si-RNA-SFRP4 led to an increase in epithelial growth (**E**) whilst the same treatment on HGF constructs resulted in no epithelial changes compared to control, scrambled si-RNA (**F**). All images used with permission from Wiley [2]. H&E: haematoxylin and eosin; ** denotes *p*-value < 0.01.

Canonical Wnt signalling is a critical molecular pathway involved in epidermal–dermal interactions [50], thus providing the basis for investigating the contribution of Wnt signalling in the regulation of dentogingival epithelia. To test this, we treated 3D organotypic models containing HGFs with Dkk1, an antagonist of the canonical Wnt pathway [2]. We observed that treatment of the HGF constructs with 500 ng/mL rhDKK1 over 8 days resulted in minimal epithelial growth (Figure 4A,B), in contrast to the control HGF construct (without the DKK1 treatment) which showed a multilayered epithelium [2]. This finding suggests the involvement of canonical Wnt signalling in the regulation of the epithelium.

To assess the differential effects on the HGFs or HPDLFs, our lab investigated a range of Wnt agonists (Wnt1 and Wnt3) and Wnt antagonists (SFRP1 and SFRP4). We demonstrated a significant increase in mRNA expression of *SFRP4* in HPDLFs compared to HGFs, and these findings were reinforced by the immunohistochemical data, which showed the specific and abundant proteomic expression of SFRP4 in the periodontal ligament tissues [2]. SFRP4, was therefore, highly specifically expressed in HPDLFs (Figure 4C,D).

Subsequent analyses via transient siRNA knockdown of SFRP4 in HPDLF constructs led to an upregulation of epithelial growth [2]. Specifically, a 72 h knockdown with SFRP4-siRNA resulted in an increase in epithelial thickness, compared to the minimal epithelial growth observed in the control HPDLF constructs (Figure 4E). Importantly, SFRP4-siRNA-treated HGF constructs resulted in no change in epithelial growth compared to the untreated control. Thus, our findings support the presence of an inhibitory signal within HPDLF (Figure 4F) [2]. The differential mRNA expression of *SFRP4* in HPDLFs also aligns with our previous immunohistochemical data relating to HGF/HPDF constructs. HPDLFs, which expressed the inhibitory signal, SFRP4, in abundance, were associated with an atrophic epithelial phenotype, while HGFs, which expressed SFRP4 at relatively low levels, was responsible for a multilayered epithelium (Figure 3).

## 4. Epithelial Response to Mucosal Wounding and the Edge Concept

The apical migration of the JE in periodontitis could be considered a natural biological reaction to wound healing by virtue of the *edge* concept. The theory describes the change in epithelial behaviour following contact with deeper (healthy) connective tissues [51]. It centres on the notion that the boundary edge provides a trigger for epithelial migration by removing spatial constraints, allowing cells already primed for mobility to migrate into a new area without the physical barriers, as demonstrated in corneal epithelial cell sheets [52]. However, the apical migration of cells halts when it comes into contact with the DCT, in much the same way that epidermal cell movement terminates upon contact with healthy collagenous bundles [53].

**Figure 5:**
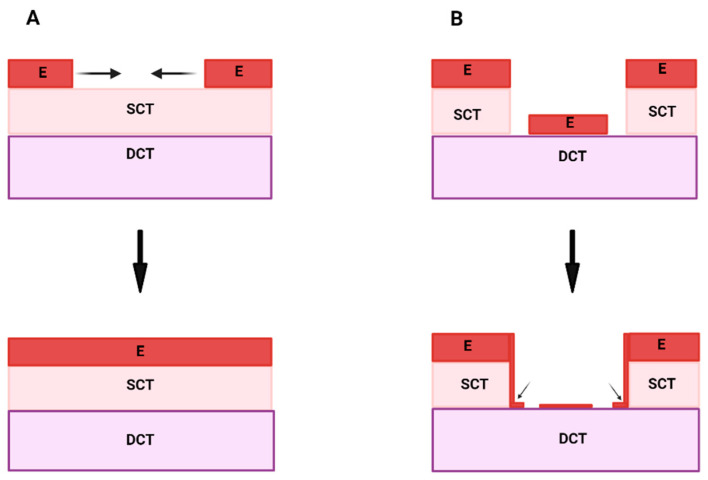
Diagram showing the epithelial response to mucosal wounding. Following superficial wounding to the mucosa in the SCT, the epithelium predictably heals over (**A**). If a deep wound is created (**B**), the epithelium will migrate apically and halt upon contact with the DCT (**B**). This edge of epithelium (arrowed) acquires a new phenotype due to the stimulus from the DCT. SCT = superficial connective tissue; DCT = deep connective tissue; E = epithelium.

The spatial configuration of epithelial and CT components following wounding elegantly models the physiological tissue configuration within the DGJ. Importantly, the nature of the epithelial response to wounding reflects the alterations to the specific underlying connective tissue components (SCT or DCT) following wounding [12,13]. If the mucosal wound is superficial and limited to the SCT, the epithelium regenerates over the wound predictably (Figure 5A).

By contrast, the epithelial response following wounding to the DCT (Figure 5B) is quite fascinating: the epithelium differentiates normally over the uninjured SCT but at either end of the wound, it migrates apically. Upon contact with the DCT at the ‘edge’, the epithelium ceases to migrate further. Therefore, the *edge* concept describes the phenomenon where the epithelium contacts the DCT following wounding [10]. This new edge of epithelium (arrowed in Figure 5B) grows a short distance onto the DCT, but further outgrowth does not occur. The new edge of the epithelium takes on an atrophic phenotype, similar to that of the JE, in health. During periodontitis, however, changes in the quality of the underlying connective tissues means that the DCT-like phenotype is no longer in proximity with the JE. Therefore, such a shift in the quality of the CT might be responsible for the alterations in the JE phenotype, as observed in periodontitis.

## 5. Development of the In Vitro Model of the DGJ

**Figure 6:**
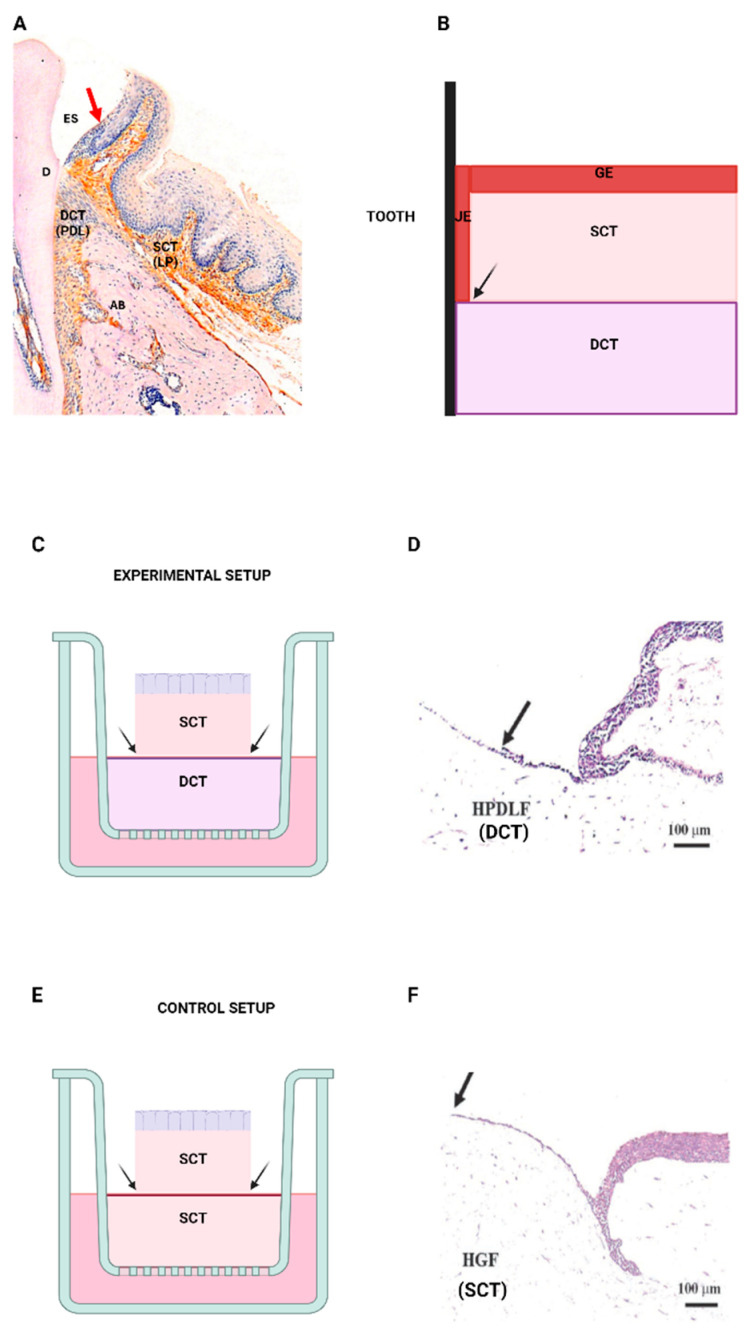
The *edge* concept. H&E section of the normal dentogingival tissues in health (**A**), the junctional epithelium is arrowed in red. Image used with permission from Wiley [2]. Schematic showing the spatial relationships of the different connective tissue components relative to the dentogingival epithelia (**B**). The gingival lamina propria, as an SCT, supports gingival epithelium, while the PDL, as a DCT is associated with the development of an atrophic epithelial phenotype, resembling the JE. The interface where the epithelium meets the DCT is known as the edge (arrowed). The anatomical relationship could be modelled in vitro, using organotypic cultures consisting of an SCT construct overlying a DCT collagen–fibroblast matrix (**C**). The larger DCT collagen bed enables the formation of an edge, where the epithelial cells would come in contact with the DCT (arrowed in C). Corresponding H&E section showing the abrupt termination of epithelial growth following contact with HPDLFs (arrowed in (**D**)). Image used with permission from Wiley [45] The control setup consisted of SCT construct supported by SCT; the arrows point to where the epithelial cells would contact the DCT (**E**). Corresponding histological section showing the continuous epithelial growth along the length of HGF construct, until the point where it ceases to migrate (arrowed) (**F**). Image used with permission from Wiley [45]. H&E: haematoxylin and eosin, SCT: superficial connective tissues, DCT: deep connective tissues.

An in vitro 3D organotypic model of the DGJ was constructed to understand the unique interactions between different tissue types within the DGJ. The in vitro DGJ model is composed of an HGF construct, and supported by a much larger collagen bed, embedded with HPDLFs. This specific arrangement was designed to reflect the distinctive anatomical, spatial configuration between the epithelial and different CT components of the DGJ (Figure 6A,B) [54]. A unique feature of the model is the creation of the boundary *edge*—the interface between HGFs and the larger HPDLF recipient collagen matrix. Epithelial cells, seeded on the smaller HGF collagen matrix, migrate apically along the sides of the collagen matrix to populate the larger HPLDF collagen bed. Therefore, the creation of the boundary edge between HGFs and HPDLFs enables phenotypic changes in the epithelium to be observed [54].

To characterise the phenotypic changes at the boundary edge, epithelial growth was quantified by measuring the length of epithelial growth along the recipient collagen matrix. The changes in epithelial phenotype (migration and proliferation) at the edge were assessed by quantifying the horizontal outgrowth on the recipient collagen matrix [54]. Using this model, we showed a significant reduction in horizontal outgrowth following contact with HPDLF collagen (Figure 6C,D), compared to the relatively longer epithelial outgrowth on the HGF collagen (control, Figure 6E,F) [54]. The data support the notion that HPDLFs do not provide the permissive signals for epithelial growth. Thus, using the in vitro DGJ model, we demonstrated significant changes to the epithelial phenotype in response to changes in the fibroblast components at the boundary edge.

This unique in vitro organotypic construct of the DGJ has the potential to serve as a plausible model for understanding the fibroblast regulation of the junctional epithelium in health and disease. The model could also be used to investigate the epithelial migration and proliferation following wounding, host microbiome interactions, testing of therapeutic interventions on the dentogingival tissues, and ageing studies of the periodontium.

## 6. Experimental Protocols

### 6.1. Three-Dimensional Organotypic Culture Modelling of Oral Tissues

**Figure 7:**
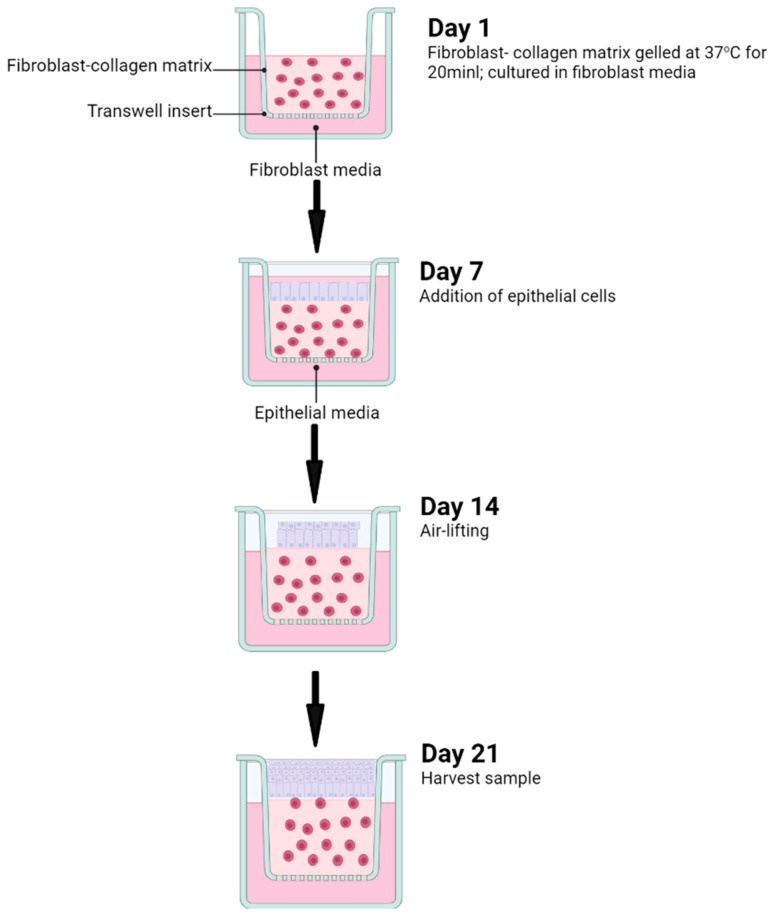
Diagram showing the experimental sequence and timing in constructing the 3D organotypic culture system. The protocol begins with the formation of the fibroblast–collagen matrix, followed by the addition of epithelial cells at day 7, air lifting at day 14 and harvesting of the samples for processing at day 21.

The 3D organotypic culture model described here consists of an epithelial component, supported by fibroblasts embedded in a collagen matrix (connective tissue equivalent). The epithelial cells and fibroblasts are initially cultured separately in monolayer (2D) cultures. However, for the 3D co-culture, each of these cellular components are brought together into one single system (Figure 7). The experimental setup begins with the culture of the fibroblasts, that are typically incorporated within the collagen gel, which undergoes contraction over time. The epithelial cells are then seeded over the fibroblast–collagen matrix and allowed to proliferate over time.

The collagen matrix, which consists of native collagen type I, is a widely used hydrogel scaffold in 3D culture systems [55], as it serves as the primary structural protein within the ECM [56]. The structural characteristics of the collagen gel can be modified to suit the experimental requirement. For instance, adjustments such as changes in pore, size, ligand density and stiffness can be achieved by varying collagen concentration or introducing chemical bonding agents. However, one limitation of collagen-based scaffolds is their relatively poor biostability, leading to a higher rate of biodegradation. Glutaraldehyde cross-linked collagen effectively modifies the biodegradation rate of collagen-based scaffolds while maintaining biocompatibility [57,58,59].

#### 6.1.1. H400 Epithelial Culture

The H400 oral epithelial cell line (ECACC 06 092 006) is composed of immortalised keratinocytes originating from a human oral alveolar cancer cell line [60]. These cells were maintained in Dulbecco’s Modified Eagle Medium/Nutrient F-12 HAM (DMEM F-12; Sigma, Burlington, MA, USA) supplemented with 10% foetal calf serum (FCS; Sigma), 4 mM L-Glutamine (Sigma), 25 µg/mL hydrocortisone (Sigma), 10 µM sodium pyruvate (Sigma) and 1% penicillin/streptomycin (pen-strep, Sigma).

#### 6.1.2. Fibroblast Culture

Human gingival fibroblasts (HGFs) were procured commercially (Sciencell Laboratories HGF #2620, that had produced a cell line pooled from two distinct donors). HGFs were cultured using reagents recommended by Sciencell Laboratories: fibroblast basal media, 2% FCS, 1% fibroblast growth supplement and 1% pen-strep (Sigma).

Human periodontal ligament fibroblasts (HPDLFs) were obtained from cultures previously isolated from PDL harvested from the tooth roots of different donors. HPDLFs were cultured in αMEM (Sigma), 10% FCS (Biosera, UK), 10 mM HEPES buffer (Sigma), 2 nM l-glutamine (Invitrogen, Waltham, MA, USA) and 1% pen-strep (Sigma).

The following protocol describes the construction of 3D organotypic cultures, created by seeding of H400 epithelial cells on type I collagen gel enriched with either HGFs or HPDLFs (Figure 7).

#### 6.1.3. Preparation of the Collagen Solution

Rat tail collagen solution was reconstituted by addition of 0.1% acetic acid (Sigma) to 10 mg vial of lyophilised collagen type I (Sigma). The solution was allowed to dissolve completely over 24 h at room temperature.

#### 6.1.4. Embedding of Fibroblasts in Collagen Gel (Day 1)

To prepare the collagen gels, the following reagents were prepared on ice: 1-part FBS (Sigma), 1-part 10×DMEM (Gibco, Waltham, MA, USA) and 8-part collagen solution (4 mg/mL, Thermofisher, Waltham, MA, USA). 10×DMEM and collagen solution were pipetted into 24-well Transwell inserts (Corning, Corning, NY, USA) and neutralised with 1 nM sodium hydroxide (NaOH) (Sigma) until the solution turned pink permanently. Finally, 2.5 × 10^5^ fibroblasts suspended in 1-part FBS were added to the DMEM collagen solution.

The collagen-fibroblast solution was incubated at 37 °C for 20 min until gelation, after which 200 µL MEM fibroblast medium was added into the Transwell to cover the collagen gel. Approximately 600–650 µL MEM was pipetted into the external culture well and the entire construct was maintained for 7 days at 37 °C and 5% CO_2_.

#### 6.1.5. Epithelial Cell Seeding (Day 7)

Following removal of the fibroblast medium from the Transwell and cell culture well, 10^5^ epithelial cells in 200 µL epithelial media were seeded over the collagen-fibroblast matrix. The constructs were submerged and cultured for 7days.

#### 6.1.6. Airlifting and Harvesting (Day 14)

The constructs were then airlifted by removing the medium from the epithelial surface and maintained for an additional 7 days. The media, which was added external to the Transwell, was replenished every 3 days until the samples were harvested at day 21, for processing.

### 6.2. Three-Dimensional Organotypic Model of the DGJ

**Figure 8:**
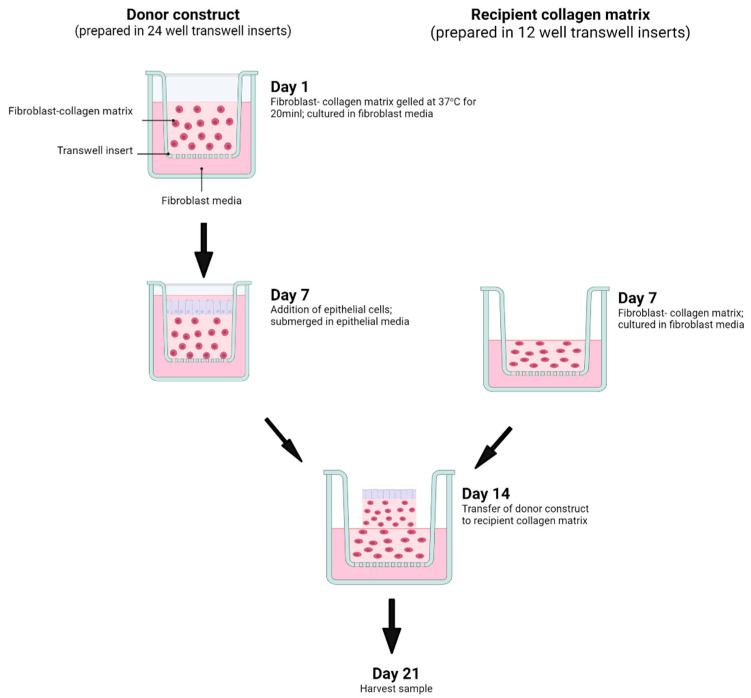
Diagram showing the experimental sequence and timing in constructing in vitro model of the dentogingival junction (DGJ). The protocol begins with the setup of the donor construct, followed by the addition of the epithelial cells at day 7. The larger recipient construct is prepared also at day 7, followed by the transfer of the donor construct to the recipient collagen matrix at day 14. The specimen is harvested at day 21.

The 3D organotypic model of the DGJ consisted of two components: a donor construct that is supported by a recipient collagen matrix. An important aspect of the protocol was the sequence and timing of the preparation for the respective HGF/HPDLF constructs, thus ensuring that both organotypic cultures were at the appropriate stage for crucial transfer step in the construction of the DGJ model. The following experimental protocol details the preparation of the DGJ construct (Figure 8).

#### 6.2.1. Preparation of the Donor HGF Construct (Day 1)

The donor HGF construct consisting of H400 epithelial cells cultured over collagen gel embedded in HGFs, was prepared in 24 well Transwell inserts, as described (in Section 6.1).

#### 6.2.2. Preparation of the Recipient HPDLF Collagen Gel (Day 7)

At day 7, the recipient fibroblast-collagen matrix was prepared by embedding 5 × 10^5^ fibroblasts with previously neutralised collagen solution and pipetted into 12-well Transwell inserts and maintained in MEM fibroblast medium for 7 days.

#### 6.2.3. Transfer Stage (Day 14) and Harvesting (Day 21)

On day 14, the donor constructs were transferred onto the recipient collagen matrices. This transfer process involved the removal of the culture medium from the Transwell, followed by the release of the construct from the walls of the Transwell using a No. 15 scalpel, then finally transferring the HGF construct onto the recipient collagen gel using a sterile microspatula. The epithelial culture medium was added to the level approximating that of the recipient collagen matrix and maintained for an additional 7 days at 37 °C and 5% CO_2_. The medium was refreshed every 3 days until the samples were harvested on day 21 and fixed for 24 h in a 10% neutral buffered formalin solution.

## 7. Limitations and Directions for Future Research

There are several limitations with the in vitro organotypic construct, which could be an over-simplification of the DGJ. Importantly, the existing co-culture lacks the presence of immune or inflammatory cellular components, and therefore, the host response element, which is integral to the pathogenesis of periodontitis [61], is absent from the in vitro model. To this end, it is important to acknowledge that the downward migration of the junctional epithelium is also a response to significant inflammatory cellular infiltrate within the stroma. Therefore, immune and inflammatory cells should be incorporated within the model to reflect the aberrant host response, which plays an integral role in the pathogenesis of periodontitis [61]. Other limitations include the absence of key ECM components in the collagen matrix such as hyaluronan and chondroitin; the absence of hemidesmosome proteins, such as Integrin α6β4 and collagen XVII, which are necessary for the reformation of the JE [62]. These ECM components, which are necessary for the structural support for the connective tissues, provide essential cues for the maintenance of adjacent tissues that deteriorate during periodontitis. Therefore, it is necessary to design an in vitro model incorporating ECM to reflect the effects of the disease on the disintegration of these cellular components.

It is also important to note the significant reduction in collagen integrity when maintained over time. To this end, the user could consider commercially available gingival mucosa (e.g., EpiOral^TM^ or EpiGingival^TM^ from MatTek, Ashland, MA, USA, as discussed in Section 3). These decellularised matrices allow for the addition of cellular components at the initiation of the assay. Furthermore, the existing co-culture model also lacks endothelial cells, human α-defensins and the antimicrobial product LL-37 (produced by the degradation of hCAP18), present within the JE [63]. Therefore, future research should focus on developing vascularised models of the DGJ, with the inclusion of endothelial, specific immune cells and even senescent fibroblasts to better mimic the chronic nature of periodontitis.

To increase the throughput in the production of these models, future research should also explore 3D bioprinting for the cellular scaffolds [64], which would enhance consistency, and provide therapeutic avenues for periodontitis [65]. In parallel with this, the incorporation of this model within organ-on-a-chip, (e.g., to model the periodontal soft tissues) should also be explored to enable the study of cellular phenotypes and interactions that better mimic the in vivo scenario [66].

## 8. Conclusions

In conclusion, this paper proposes a feasible in vitro 3D co-culture model of the DGJ to demonstrate the differential fibroblast influences in the maintenance of the dentogingival epithelia. This unique model, which is the first of its kind to model the DGJ, features the boundary edge between the HGF- and HPDLF-collagen matrices, thus enabling differences in epithelial migration and proliferation to be observed. Importantly, the paper comprehensively details the experimental protocol for the construction of the in vitro organotypic culture, that can be utilised in a variety of applications in periodontal research to enhance our understanding of wounding, host microbiome interactions, pathogenesis, ageing and the specific effects of therapeutic interventions on the dentogingival epithelia.
